# Supplementary material for: Damselfishes alleviate the impacts of sediments on host corals
Source: R Soc Open Sci. 2020 Apr 29;7(4):192074. doi: 10.1098/rsos.192074 (PMC7211878; doi:10.1098/rsos.192074)
Supplement: Electronic Supplementary Material [file rsos192074supp1.pdf]

The following supplement accompanies the article

Damselfishes alleviate the impacts of sediments on their host corals

---

Supplemental Text S1: Field sediment quantification

Methods

Field sediment methods

Field sampling was conducted between April – June 2017 around the Palm Islands, an inner-shelf, island group of the Great Barrier Reef (GBR). Orpheus Island is located ~20 km from the Queensland coast and close to the Herbert (20 km) and Burdekin rivers (150 km). Sediments were collected at six locations around the Palm Islands to quantify and identify natural levels of sedimentation in the area pertinent to the aquaria experiment (Figure S1). Sediment traps were deployed from three exposed sites (SE Pelorus, East Orpheus, Snapper Point), and three sheltered sites (SW Pelorus, Cattle Bay, Hazard Bay), over the course of 34 days, roughly 3 months post-wet season.

Quantification of sediment dynamics

Sediment accumulation was measured using simple tube traps constructed from PVC. Sediment trap design and placement followed trap design recommendations [1]. Specifically, the dimensions of traps were as follows: trap diameter (D): 54 mm; trap height (H): 30 mm, with a 5 cm diameter mesh placed in the top of the trap to limit resuspension, or organisms getting inside the trap. Traps were affixed to a picket on the reef ensuring they were vertical. The cylindrical traps had a diameter greater than 50mm and a trap Reynolds number (R_t) of ~6:1. Twenty-four traps, four per site, deployed at depths between 2-5.5 m, lowest astronomical tide (LAT), and were positioned with the sediment trap mouth at ~70 cm above

the reef substrate. Traps were deployed three times per site, each deployment lasting 7-13 days.

In addition to traps, at a sub-sample of 4 sites, sediment samples from the epilithic algal matrix (EAM) were collected as a third measure of sedimentation/accumulation. Using an underwater sediment vacuum apparatus (submersible 12 V electron vacuum sampler, design [2,3]), all sediment was removed from a defined area of 78.5 cm^2 (circular pipe with a 10 cm diameter) of EAM. Suitable EAM covered areas were approximately horizontal, flat (i.e. free of holes or sediment retaining pits), free of macroalgae and encrusting organisms, and covered by algal turfs [4]. Subsequently, 12 - 31 days later the areas were vacuumed again and the sediments were retained to assess accumulation rate during this time period.

Sample processing

All collected sediments from traps and vacuum samples were frozen and transported to James Cook University for further processing. Sediment samples were then rinsed with fresh water three times to remove salts, transferred into labelled sample containers, dried at 60°C (Axyos Microdigital Incubator) for > 4 days, weighed for constant weight (g), sieved into three factions [5]: <125 µm (very fine sand and silt), 125-500 µm (fine to medium sand), 500-4000 µm (coarse sand to gravel) and weighed (using Kern PCB, John Morris Scientific balance, precision 0.001 g). All sediment samples were converted to mg cm^2 day^{-1} for consistency.

Results

Sediment around the Palm Islands results

Sedimentation rates measured using sediment traps varied markedly between exposed and sheltered locations ranging from 2 – 1982 mg cm^{-2} day^{-1}. Total average daily sediment

load around the Palm Islands (all sites pooled) was ~140 mg cm^{-2} day^{-1}, with sheltered sites averaging 7 mg cm^{-2}, day^{-1}, and exposed sites averaging 300 mg cm^{-2} day^{-1}., Two of the six sites had average sedimentation values over 100 mg cm^{-2} day $^{-1}$, and >100 mg cm^{-2} day^{-1} was recorded in samples at four sites. Southeast Pelorus recorded the highest sedimentation values via sediment traps at 1062 ± 9.3 mg cm^{-2} day^{-1}). Sediment accumulation rates in the algal turfs of three sheltered and one exposed site (Snapper Point) recorded lower average sediment deposition than sediment traps (table S1); of these algal turf samples, Snapper Point displayed ten-fold more vacuumed sediments (18.8 ± 9.3 mg cm^{-2} day^{-1}) than sheltered sites.

Discussion

The sedimentation rates and grain size distributions quantified around the Palm Islands, varied markedly among habitats and the methods used. This supports a number of previous studies that have noted differences in sediment dynamics among habitats [4,6,7] and quantification methods [1,8]. The hydrodynamic activity that suspended medium and coarse sediments on exposed sites [9,10] may lead to higher coral abrasion rates in these habitats [11–13]. By contrast, lower-energy hydrodynamic activity in sheltered locations can lead to coral smothering, characteristic of inshore GBR reefs [14].

While sediment traps have been criticised for measuring a trapping rate as they limit resuspension activity [1] and the vacuum samples were used to mimic sediment dynamics on algal turf covered surfaces [4], the most accurate representation of sediment deposition on our focal branching corals is probably achieved by using a combination of both techniques. This may due to the fact that many corals have exposed flat coral surfaces (e.g. massive *Porites* or exposed areas of branching corals), as well as intricate branch structures where hydrodynamic activity is reduced, thus facilitating sedimentation and limiting resuspension (as in traps). However, data pertaining to the vacuum sediment samples should be interpreted

with caution as only four replicates per site were deployed (with only one exposed site, due to sampling conditions) and re-sampling ranged between 12-31 days. Such extended periods of time between sampling may have allowed for the EAM to saturate with sediments (with remaining sediment being resuspended or transported away), thus leading to conservative estimates of deposition rates. In the aquarium sediment deposition experiment, we spread 100 mg cm^{-2} day^{-1} on *P. damicornis* for relative comparison to the field conditions.

Literature cited

1. Storlazzi CD, Field ME, Bothner MH. 2011 The use (and misuse) of sediment traps in coral reef environments: Theory, observations, and suggested protocols. *Coral Reefs* **30**, 23–38. (doi:10.1007/s00338-010-0705-3)
2. Purcell SW. 1996 A direct method for assessing sediment load in epilithic algal communities. *Coral Reefs* **15**, 211–213. (doi:10.1007/BF01787453)
3. Kramer MJ, Bellwood DR, Bellwood O. 2012 Cryptofauna of the epilithic algal matrix on an inshore coral reef, Great Barrier Reef. *Coral Reefs* **31**, 1007–1015. (doi:10.1007/s00338-012-0924-x)
4. Tebbett SB, Goatley CHR, Bellwood DR. 2017 Algal turf sediments and sediment production by parrotfishes across the continental shelf of the northern Great Barrier Reef. *PLoS One* **12**, 1–17. (doi:10.1371/journal.pone.0170854)
5. Wentworth C. 1992 A scale of grade and class terms for clastic sediments. *J. Geol.* **30**, 377–392. (doi:10.1086/622910)
6. Purcell SW. 2000 Association of epilithic algae with sediment distribution on a windward reef in the northern Great Barrier Reef, Australia. *Bull. Mar. Sci.* **66**, 199–214.
7. Browne NK, Smithers SG, Perry CT. 2013 Spatial and temporal variations in turbidity

on two inshore turbid reefs on the Great Barrier Reef, Australia. *Coral Reefs* **32**, 195–210. (doi:10.1007/s00338-012-0965-1)

8. Whinney J, Jones R, Duckworth A, Ridd P. 2017 Continuous *in situ* monitoring of sediment deposition in shallow benthic environments. *Coral Reefs* **36**, 521–533. (doi:10.1007/s00338-016-1536-7)
9. Yahel R, Yahel G, Genin A. 2019 Daily cycles of suspended sand at coral reefs: a biological control. *Limnol. Oceanogr.* **47**, 1071–1083.
10. Weber M, Lott C, Fabricius KE. 2006 Sedimentation stress in a scleractinian coral exposed to terrestrial and marine sediments with contrasting physical, organic and geochemical properties. *J. Exp. Mar. Bio. Ecol.* **336**, 18–32. (doi:10.1016/j.jembe.2006.04.007)
11. Loya Y. 1976 Effects of Water Turbidity and Sedimentation. *Bull. Mar. Sci.* **26**, 450–466.
12. Rogers C. 1990 Response of coral reefs and reef organisms to sedimentation. *Mar. Ecol. Prog. Ser.* **62**, 185–202. (doi:10.3354/meps062185)
13. PIANC. 2010 *Dredging and port construction around coral reefs. Report No. 108.*
14. Fabricius KE. 2005 Effects of terrestrial runoff on the ecology of corals and coral reefs: Review and synthesis. *Mar. Pollut. Bull.* **50**, 125–146. (doi:10.1016/j.marpolbul.2004.11.028)

Supplemental figures and tables associated with sediment sampling

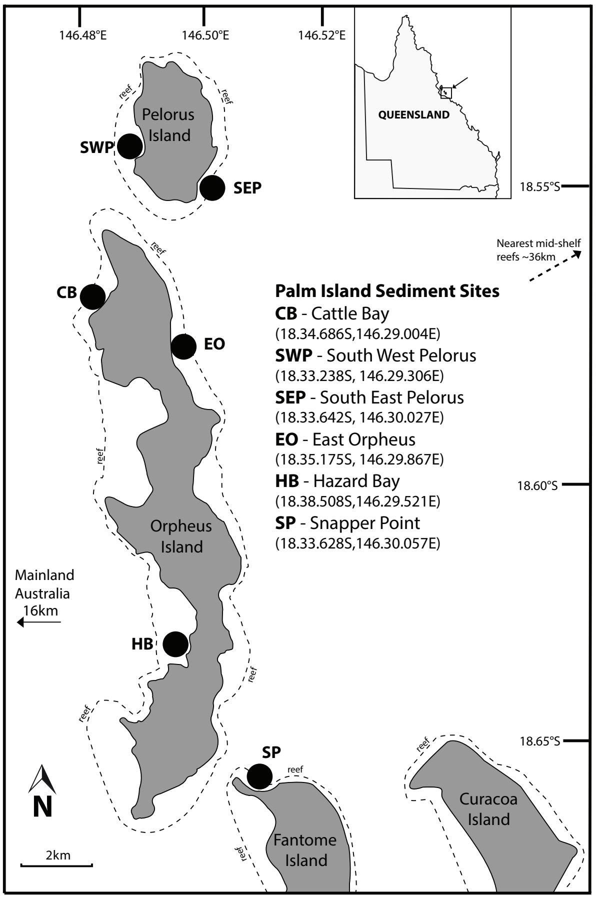

Figure S1. Sampling locations around the Palm Island reefs, located ~12 km from the Queensland coast. SWP, CB, and HB sites are sheltered locations, on the leeward side of Pelorus and Orpheus Island, and SEP, EO, and SP are exposed locations on the windward side of Pelorus, Orpheus, and Fantome Islands. Two sediment traps were deployed three times per site, at depths of 2-5.5 m (LAT), from May to June 2017.

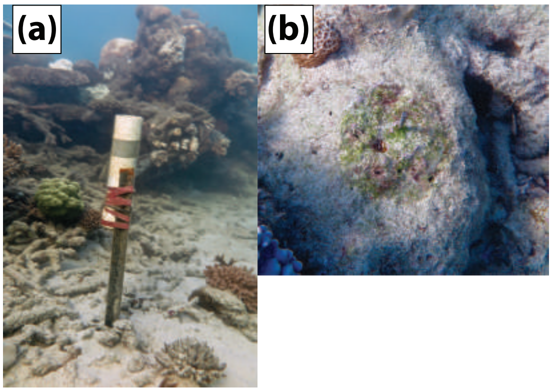

Figure S2. Example methods and deployment of sediment capture in the field: **(a)** sediment trap and **(b)** sediment vacuum collection area.

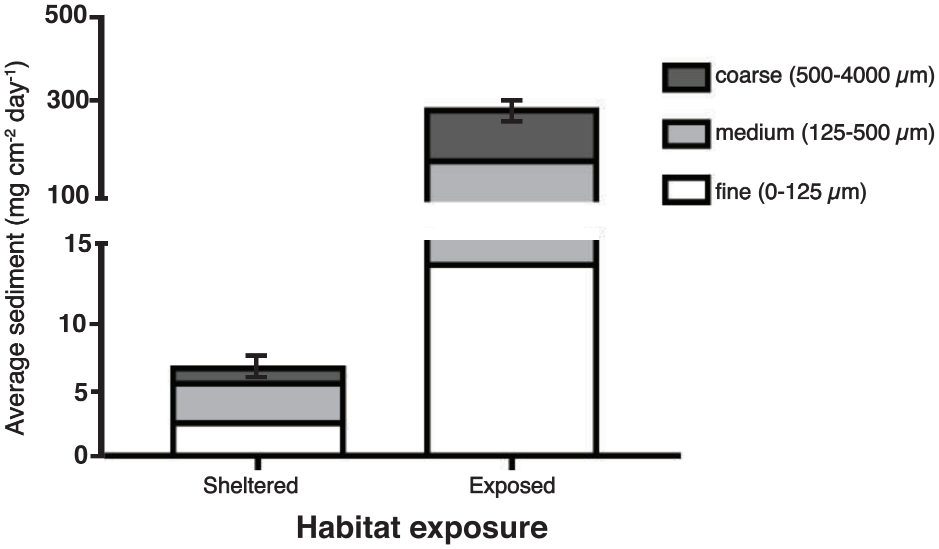

Figure S3. Sedimentation rates measured using traps on sheltered (n = 36) and exposed sites (n = 30) sites around the Palm Islands. Sediment traps deployed per site were as follows: Cattle Bay (n = 12), Hazard Bay (n = 12), SW Pelorus (n = 12), SE Pelorus (n = 7), Snapper Point (n = 12), East Orpheus (n = 11).

Table S1 Average sediments (mg cm^{-2} day^{-1}) ± S.E at four locations around the Palm Islands, collected by a submersible vacuum.

**Site**	**Exposure**	**Collections**	**Average sediment** (mg cm^{-2} day^{-1}) ± S.E
SW Pelorus	*sheltered*	4	1.93 ± 0.5
Cattle Bay	*sheltered*	4	1.85 ± 0.5
Hazard Bay	*sheltered*	4	4.83 ± 1.6
Snapper Point	*exposed*	4	18.76 ± 9.3

Supplemental Text S2 – Extended methods and results for experimental study

Methods

Suspended sediment samples were collected 1 h after sediment was added, using a syringe placed 5 cm above the coral colony. This sampling was conducted to determine if fish re-suspended sediment. Suspension samples were filtered using Whatman glass fibre filter paper (MicroScience, MSGA grade, 47 mm) and vacuum (John Morris Air Admiral), dried and weighed (using a Sartorius Entris 124I-1S, precision 0.0001 g). Suspended sediment in aquaria was analysed with a two-way ANOVA with sediment and fish as fixed factors. Again, model assumptions were assessed with residual plots.

Results

Suspended sediment concentrations were negligible in aquaria ($<6.2 \times 10^{-5}$ mg ml^{-1}, see Table S2 and Figure S3) one hour after sediment dosage. Indeed, water in the aquaria appeared clear and comparable with non-sediment treatments. Suspended sediments did not differ significantly between sediment treatments ($F_{1,46} = 0.193$, $p = 0.662$) nor with fish treatments ($F_{2,48} = 2.634$, $p = 0.082$).

Supplemental table and figures associated with the sediment experiment

Table S2. Descriptions and mineral compositions of sorted sediment fractions [1,2]. All sediments had zero microbial content.

Location	Size class	Mineral description and XRD analysis
Middle Reef, inshore GBR	<53µm	Grey-brown silt
Davie's Reef, mid-shelf GBR	<53 µm	Carbonate sediments<br>white silt, carbonate: 80% aragonite, 20% calcite,<br>Total Organic Carbon: 0.27%
Orpheus Island, inshore island GBR	90-4000 µm	Coarse beach sediments<br>tan-brown subangular to rounded to bioclastics sand: 45% quartz, 19% kaolinite, 7% albite, carbonate: 30% Calcite<br>Total organic Carbon: 2.76%

1. Esslemont G. 2000 Heavy metals in seawaters, marine sediments and corals from the Townsville section, Great Barrier Reef Marine Park, Queensland. *Mar. Chem.* **71**, 215-231. (doi:10.1016/S0304-4203(00)00050-5)
2. Ricardo GF, Jones RJ, Clode PL, Negri AP. 2016 Mucous secretion and cilia beating defend developing coral larvae from suspended sediments. *PLoS One* **11**, e0162743. (doi:10.1371/journal.pone.0162743)

Table S3. Two-way analysis of variance (ANOVA) results table for average suspended sediments (mg ml^{-1}) under different fish and sediment treatments in the manipulated sediment experiment.

Source	DF	SS	MS	F-value	P
Sediment	1	2.2×10^{-10}	2.16×10^{-10}	0.193	0.663
Fish	2	5.78×10^{-9}	2.89×10^{-9}	2.581	0.087
Sediment*Fish	2	1.15×10^{-9}	5.78×10^{-10}	0.515	0.601
Residuals	46	5.14×10^{-8}	1.12×10^{-9}		

Table S4. Tukey's HSD *post-hoc* multiple comparisons table (including confidence intervals) for total sediment, log (x+1) transformed, left on experimental *P. damicornis* colonies in the manipulative sediment experiment. Only 'sediment added' treatment colonies were included in the analysis and subsequent Tukey HSD post-hoc tests, as all colonies in the 'no sediment' treatment exhibited very low (<0.3 g) sediment accumulation over 28 days in aquaria.

Treatment comparison	Lower	Upper	P adjusted
Sediment *P. moluccensis* – Sediment *D. aruanus*	0.2714	1.36110	**0.0023**
Sediment No fish – Sediment *D. aruanus*	1.1231	2.2128	**<0.0001**
Sediment No fish – Sediment *P. moluccensis*	0.3069	1.3966	**0.0015**

Table S5. Results of pairwise tests following the permutational analysis of multivariate dispersions (PERMDISP), which assessed the homogeneity of sediment grain size distributions remaining on coral colonies under different fish treatments (*D. aruanus*, *P. moluccensis*, and No fish) in the manipulated sediment experiment. Only data from coral colonies with sediment added were included.

PERMDISP: $F_{2,33} = 15.699$, p = **0.002**

Site comparison	P-adjusted
D. aruanus – *P. moluccensis*	**0.0022**
D. aruanus – No Fish	**0.0002**
P. moluccensis – No Fish	0.4277

Table S6. Results of a beta regression model examining the proportion of partial colony mortality for *P. damicornis* colonies under different sediment and fish treatments in the manipulated sediment experiment.

Factor	z-value	p-value
(intercept)	-13.427	$<2 \times 10^{-16}$
P. moluccensis	0.000	1.0000
No Fish	0.000	1.0000
Sediment added	0.893	0.3317
P. moluccensis : Sediment added	2.468	**0.0149**
No Fish : Sediment added	3.498	**0.0002**

Table S7. Results of lsmeans pairwise comparisons with a Tukey's adjustment based on a betaregression model comparing the interacting effects of sediment exposure and fish treatment on partial colony mortality rates of *P. damicornis* colonies in the experiment.

Treatment comparison	z ratio	P adjusted
Clean *D. aruanus* – Clean *P. moluccensis*	0.000	1.0000
Clean *D. aruanus* – Clean No fish	0.000	1.0000
Clean *D. aruanus* – Sediment *D. aruanus*	-0.881	0.9210
Clean *D. aruanus* – Sediment *P. moluccensis*	**-3.880**	**0.0007**
Clean *D. aruanus* – Sediment No Fish	**-5.082**	**<0.0001**
Clean *P. moluccensis* – Clean No Fish	0.000	1.0000
Clean *P. moluccensis* – Sediment *D. aruanus*	-0.894	0.9210
Clean *P. moluccensis* – Sediment *P. moluccensis*	-3.892	**0.0007**
Clean *P. moluccensis* – Sediment No Fish	**-5.090**	**<0.0001**
Clean No Fish – Sediment *D. aruanus*	**-0.894**	0.9210
Clean No Fish – Sediment *P. moluccensis*	-3.892	**0.0007**
Clean No Fish – Sediment No Fish	**-5.090**	**<0.0001**
Sediment *D. aruanus* – Sediment *P. moluccensis*	**-3.404**	**0.0060**
Sediment *D. aruanus* – Sediment No Fish	**-4.734**	**<0.001**
Sediment *P. moluccensis* – Sediment No Fish	**-1.880**	**0.0461**

Table S8. Results of multiple selected comparisons as a *post-hoc* test of the two-way analyses of variance (ANOVAs) analysing the effects of sediment and fish presence on *P. damicornis* colonies. Separate ANOVAs were completed for the two phases of the experiment: Start and End. Samples sizes for each treatment are displayed in brackets.

Coral tissue	Phase	Comparison	Lower	Upper	P-value
**Total Chlorophyll**	Start	Clean *D. aruanus* (n = 12) – Clean *P. moluccensis* (n = 12)	-0.9533	0.9082	0.9999
		Clean *D. aruanus* (n = 12) – Clean No fish (n = 12)	-0.9373	0.7861	0.9998
		Clean *D. aruanus* (n = 12) – Sediment *D. aruanus* (n = 12)	-0.9141	0.9935	0.9997
		Clean *D. aruanus* (n = 12) – Sediment *P. moluccensis* (n = 12)	0.5867	1.2208	0.9047
		Clean *D. aruanus* (n = 12) – Sediment No Fish (n = 12)	-0.6782	1.0452	0.9885
		Clean *P. moluccensis* (n = 12) – Clean No Fish (n = 12)	-0.9383	0.8777	0.9999
		Clean *P. moluccensis* (n = 12) – Sediment *D. aruanus* (n = 12)	-0.8575	1.0821	0.9994
		Clean *P. moluccensis* (n = 12) – Sediment *P. moluccensis* (n = 12)	-0.6302	1.3094	0.9905
		Clean *P. moluccensis* (n = 12) – Sediment No Fish (n = 12)	0.7247	1.1368	0.9864
		Clean No Fish (n = 12) – Sediment *D. aruanus* (n = 12)	0.7384	1.0691	0.9943
		Clean No Fish (n = 12) – Sediment *P. moluccensis* (n = 12)	-0.5110	1.2965	0.7947
		Clean No Fish (n = 12)– Sediment No Fish (n = 12)	-0.6026	1.1208	0.9485
		Sediment *D. aruanus* (n = 12) – Sediment *P. moluccensis* (n = 12)	-0.7166	1.1713	0.9801
		Sediment *D. aruanus* (n = 12) – Sediment No Fish (n = 12)	-0.8100	0.9975	0.9996

		Sediment *P. moluccensis* (n = 12) – Sediment No Fish (n = 12)	-1.0374	0.7701	0.9979
	End	Clean *D. aruanus* (n = 11) – Clean *P. moluccensis* (n = 12)	-4.4159	2.6053	0.9734
		Clean *D. aruanus* (n = 11) – Clean No fish (n = 12)	-5.1124	1.9089	0.7607
		Clean *D. aruanus* (n = 11) – Sediment *D. aruanus* (n = 10)	-2.8080	4.5414	0.9820
		Clean *D. aruanus* (n = 11) – Sediment *P. moluccensis* (n = 11)	-4.8683	2.3039	0.8983
		Clean *D. aruanus* (n = 11) – Sediment No Fish (n = 12)	-6.7723	0.2489	0.0832
		Clean *P. moluccensis* (n = 12) – Clean No Fish (n = 12)	-4.1299	2.7371	0.9909
		Clean *P. moluccensis* (n = 12) – Sediment *D. aruanus* (n = 10)	-1.8290	5.3731	0.6984
		Clean *P. moluccensis* (n = 12) – Sediment *P. moluccensis* (n = 11)	-3.8875	3.1338	0.9996
		Clean *P. moluccensis* (n = 12) – Sediment No Fish (n = 12)	-5.8875	1.0771	0.3441
		Clean No Fish (n = 12) – Sediment *D. aruanus* (n = 10)	-1.1326	6.0695	0.3454
		Clean No Fish (n = 12) – Sediment *P. moluccensis* (n = 11)	-3.1911	3.3802	0.9998
		Clean No Fish (n = 12)– Sediment No Fish (n = 12)	-5.0934	1.7735	0.7138
		Sediment *D. aruanus* (n = 10) – Sediment *P. moluccensis* (n = 11)	-5.8236	1.5258	0.5244
		Sediment *D. aruanus* (n = 10) – Sediment No Fish (n = 12)	-7.7294	0.5273	**0.0156**
		Sediment *P. moluccensis* (n = 11) – Sediment No Fish (n = 12)	-5.4901	1.5312	0.5641
**Total Proteins**	Start	Clean *D. aruanus* (n = 12) – Clean *P. moluccensis* (n = 12)	-0.6457	1.0649	0.9789
		Clean *D. aruanus* (n = 12) – Clean No fish (n = 12)	-0.7991	0.9115	0.9999

	Clean *D. aruanus* (n = 12) – Sediment *D. aruanus* (n = 12)	-0.6507	1.0599	0.9811
	Clean *D. aruanus* (n = 12) – Sediment *P. moluccensis* (n = 12)	-1.1199	0.5907	0.9431
	Clean *D. aruanus* (n = 12) – Sediment No Fish (n = 12)	-0.7522	0.9584	0.9992
	Clean *P. moluccensis* (n = 12) – Clean No Fish (n = 12)	-1.0087	0.7019	0.9949
	Clean *P. moluccensis* (n = 12) – Sediment *D. aruanus* (n = 12)	-0.8603	0.8503	1.0000
	Clean *P. moluccensis* (n = 12) – Sediment *P. moluccensis* (n = 12)	-1.3295	0.3811	0.5838
	Clean *P. moluccensis* (n = 12) – Sediment No Fish (n = 12)	-0.9618	0.7488	0.9991
	Clean No Fish (n = 12) – Sediment *D. aruanus* (n = 12)	-0.7069	1.0037	0.9957
	Clean No Fish (n = 12) – Sediment *P. moluccensis* (n = 12)	-1.1762	0.5344	0.8792
	Clean No Fish (n = 12)– Sediment No Fish (n = 12)	-0.8085	0.9021	0.9999
	Sediment *D. aruanus* (n = 12) – Sediment *P. moluccensis* (n = 12)	-1.3245	0.3861	0.5948
	Sediment *D. aruanus* (n = 12) – Sediment No Fish (n = 12)	-0.9568	0.7538	0.9993
	Sediment *P. moluccensis* (n = 12) – Sediment No Fish (n = 12)	-0.4876	1.2229	0.8044
End	Clean *D. aruanus* (n = 12) – Clean *P. moluccensis* (n = 12)	-0.1868	0.0177	0.1622
	Clean *D. aruanus* (n = 12) – Clean No fish (n = 12)	0.1656	0.0288	0.4607
	Clean *D. aruanus* (n = 12) – Sediment *D. aruanus* (n = 11)	-0.1467	0.0623	0.8423
	Clean *D. aruanus* (n = 12) – Sediment *P. moluccensis* (n = 12)	-0.1518	0.0537	0.7129
	Clean *D. aruanus* (n = 12) – Sediment No Fish (n = 12)	-0.2421	0.0376	**0.0021**

		Clean *P. moluccensis* (n = 12) – Clean No Fish (n = 12)	0.0811	0.1234	0.9901
		Clean *P. moluccensis* (n = 12) – Sediment *D. aruanus* (n = 11)	-0.0622	0.1469	0.8406
		Clean *P. moluccensis* (n = 12) – Sediment *P. moluccensis* (n = 12)	-0.0673	0.1372	0.9149
		Clean *P. moluccensis* (n = 12) – Sediment No Fish (n = 12)	-0.1575	0.0469	0.6092
		Clean No Fish (n = 12) – Sediment *D. aruanus* (n = 11)	-0.0834	0.1257	0.9911
		Clean No Fish (n = 12) – Sediment *P. moluccensis* (n = 12)	-0.0884	0.1160	0.9987
		Clean No Fish (n = 12)– Sediment No Fish (n = 12)	-0.1787	0.0258	0.2538
		Sediment *D. aruanus* (n = 11) – Sediment *P. moluccensis* (n = 12)	-0.1119	0.0972	0.9999
		Sediment *D. aruanus* (n = 11) – Sediment No Fish (n = 12)	-0.2022	0.0069	0.0807
		Sediment *P. moluccensis* (n = 12) – Sediment No Fish (n = 12)	-0.1925	0.1199	0.1139
**Tissue Biomass**	Start	Clean *D. aruanus* (n = 12) – Clean *P. moluccensis* (n = 12)	-0.4099	0.7798	0.9419
		Clean *D. aruanus* (n = 12) – Clean No fish (n = 12)	-0.5717	0.6189	0.9999
		Clean *D. aruanus* (n = 12) – Sediment *D. aruanus* (n = 12)	-0.5245	0.6652	0.9993
		Clean *D. aruanus* (n = 12) – Sediment *P. moluccensis* (n = 12)	-0.7046	0.4851	0.9942
		Clean *D. aruanus* (n = 12) – Sediment No Fish (n = 12)	-0.4528	0.7368	0.9812
		Clean *P. moluccensis* (n = 12) – Clean No Fish (n = 12)	-0.7667	0.4430	0.9668
		Clean *P. moluccensis* (n = 12) – Sediment *D. aruanus* (n = 12)	-0.7095	0.4803	0.9929
		Clean *P. moluccensis* (n = 12) – Sediment *P. moluccensis* (n = 12)	-0.8896	0.3002	0.6941

	Clean *P. moluccensis* (n = 12) – Sediment No Fish (n = 12)	-0.6378	0.5519	0.9999
	Clean No Fish (n = 12) – Sediment *D. aruanus* (n = 12)	-0.5476	0.6421	0.9999
	Clean No Fish (n = 12) – Sediment *P. moluccensis* (n = 12)	-0.7277	0.4620	0.9861
	Clean No Fish (n = 12)– Sediment No Fish (n = 12)	-0.4760	0.7137	0.9916
	Sediment *D. aruanus* (n = 12) – Sediment *P. moluccensis* (n = 12)	-0.7750	0.4148	0.9479
	Sediment *D. aruanus* (n = 12) – Sediment No Fish (n = 12)	-0.5232	0.6665	0.9992
	Sediment *P. moluccensis* (n = 12) – Sediment No Fish (n = 12)	-0.3431	0.8466	0.8145
End	Clean *D. aruanus* (n = 12) – Clean *P. moluccensis* (n = 12)	-0.0108	0.0038	0.7184
	Clean *D. aruanus* (n = 12) – Clean No fish (n = 12)	-0.0076	0.0070	0.9999
	Clean *D. aruanus* (n = 12) – Sediment *D. aruanus* (n = 11)	-0.0066	0.0083	0.9995
	Clean *D. aruanus* (n = 12) – Sediment *P. moluccensis* (n = 12)	-0.0076	0.0071	0.9999
	Clean *D. aruanus* (n = 12) – Sediment No Fish (n = 12)	-0.0122	0.0024	0.3661
	Clean *P. moluccensis* (n = 12) – Clean No Fish (n = 12)	-0.0041	0.0105	0.7881
	Clean *P. moluccensis* (n = 12) – Sediment *D. aruanus* (n = 11)	-0.0031	0.0118	0.5307
	Clean *P. moluccensis* (n = 12) – Sediment *P. moluccensis* (n = 12)	-0.0040	0.0106	0.7763
	Clean *P. moluccensis* (n = 12) – Sediment No Fish (n = 12)	-0.0087	0.0059	0.9930
	Clean No Fish (n = 12) – Sediment *D. aruanus* (n = 11)	-0.0063	0.0086	0.9977
	Clean No Fish (n = 12) – Sediment *P. moluccensis* (n = 12)	-0.0073	0.0074	1.0000

Clean No Fish (n = 12)– Sediment No Fish (n = 12)	-0.0119	0.0027	0.4381
Sediment *D. aruanus* (n = 11) – Sediment *P. moluccensis* (n = 12)	-0.0085	0.0064	0.9982
Sediment *D. aruanus* (n = 11) – Sediment No Fish (n = 12)	-0.0132	0.0017	0.2252
Sediment *P. moluccensis* (n = 12) – Sediment No Fish (n = 12)	-0.0119	0.0026	0.4249

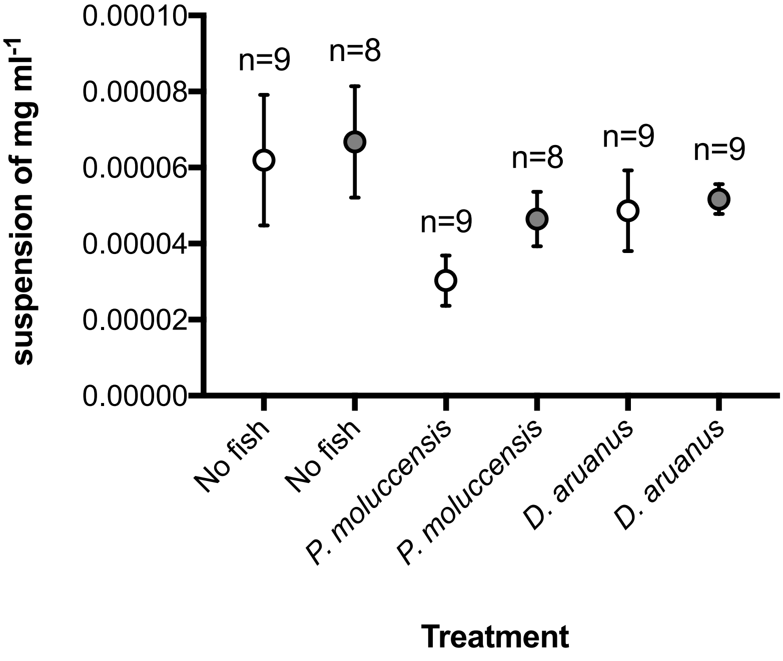

Figure S4. Average suspended sediments (mg ml^{-1}) per treatment in the manipulative sediment experiment. No sediment treatments are displayed with white dots and with sediment added are displayed with grey dots. Half of the total coral colonies were exposed to sediment treatments and treatment sample sizes analysed (n) are displayed above points.

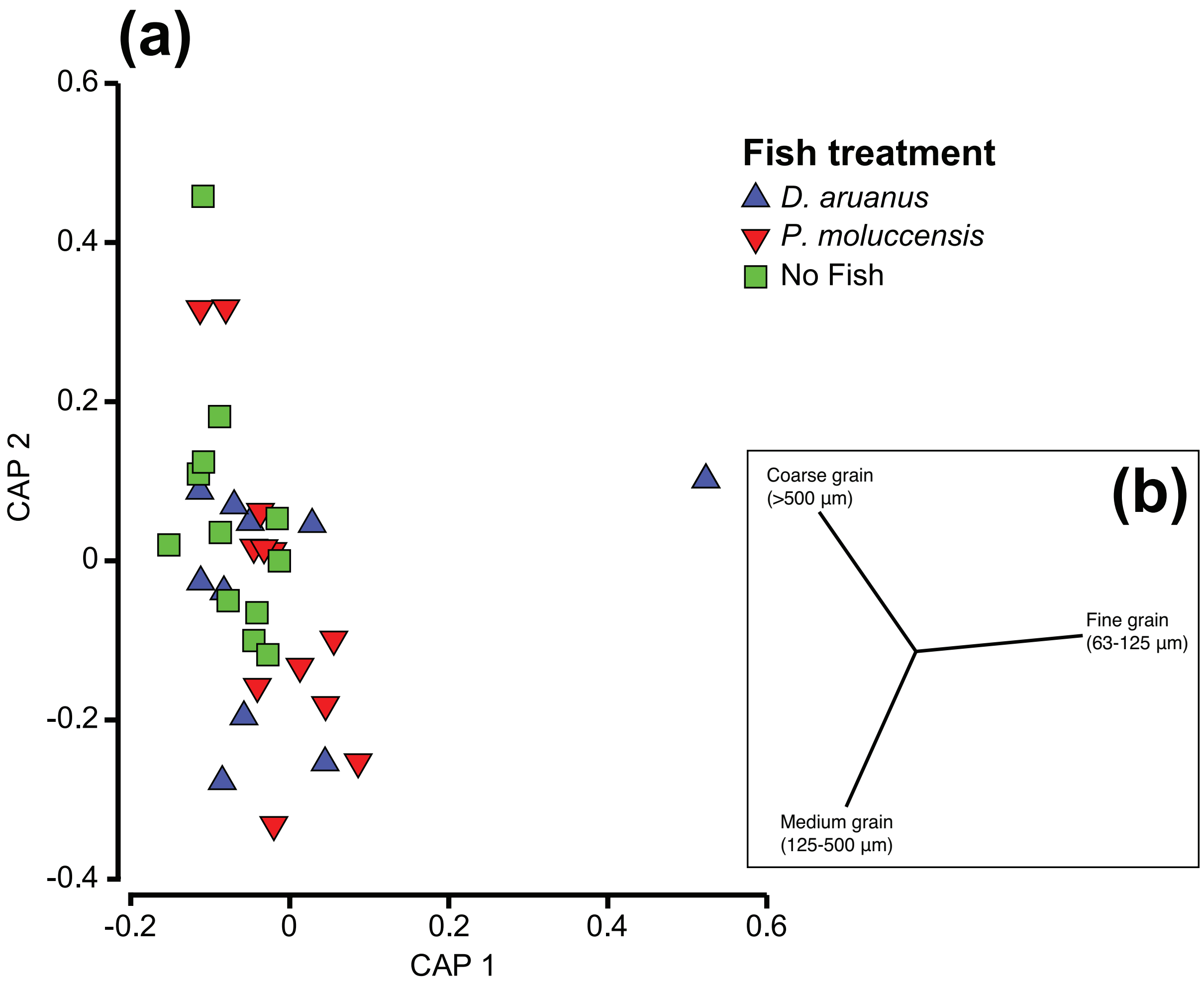

Figure S5. (a) Canonical analysis of principal (CAP) coordinates ordination based on a Euclidean distance matrix of standardised grain size distribution data from sediments remaining on coral colonies under different fish treatments (No fish, *D. aruanus*, and *P. moluccensis*). **(b)** Vectors show the relationship among sediment grain size fractions (µm) and how they influence the position of data points in the CAP. Vectors were calculated using a multiple correlation model.

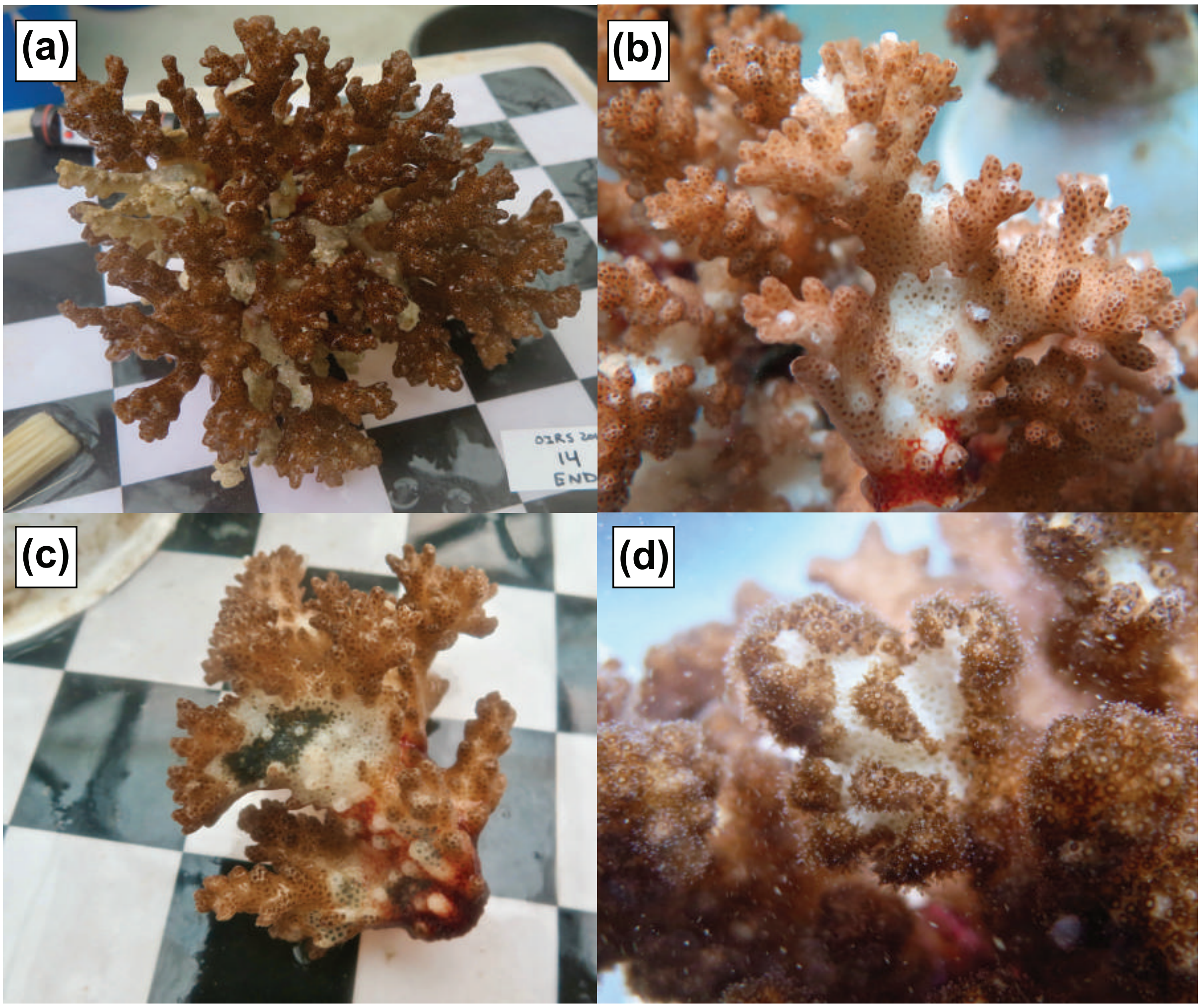

Figure S6. Sub-lethal effects of sediments on *P. damicornis* in experimental laboratory conditions. Qualitative observations of bleaching and partial mortality on **(a)** whole *P. damicornis* colonies in aquaria subjected to sedimentation rates of ~100 mg cm^{-2} day^{-1} for 28 days and **(b - d)** examples of reduced tissue health and partial mortality (tissue necrosis) in selected colony fragments.
